# Risk of Weight Loss in Adult Patients and the Effect of Staffing Registered Dietitians in Kaifukuki (Convalescent) Rehabilitation Wards: A Retrospective Analysis of a Nationwide Survey

**DOI:** 10.3390/healthcare9060753

**Published:** 2021-06-18

**Authors:** Shinta Nishioka, Yoji Kokura, Takatsugu Okamoto, Masako Takayama, Ichiro Miyai

**Affiliations:** 1 Nutrition Committee, Kaifukuki Rehabilitation Ward Association, Tokyo 101-0047, Japan; yojikokura@hotmail.com (Y.K.); mcjnutrition@juryo.or.jp (M.T.); 2 Annual Survey Committee, Kaifukuki Rehabilitation Ward Association, Tokyo 101-0047, Japan; takatsugu@amy.hi-ho.ne.jp (T.O.); miyai@omichikai.or.jp (I.M.); 3 Department of Clinical Nutrition and Food Services, Nagasaki Rehabilitation Hospital, Nagasaki 850-0854, Japan; 4 Department of Clinical Nutrition, Keiju Medical Center, Ishikawa 926-0866, Japan; 5 Nishi-Hiroshima Rehabilitation Hospital, Hiroshima 731-5143, Japan; 6 Department of Nutrition, Kumamoto Kinoh Hospital, Kumamoto 860-8518, Japan; 7 Neurorehabilitation Research Institute, Morinomiya Hospital, Osaka 536-0025, Japan

**Keywords:** body weight loss, registered dietitian, inpatient rehabilitation facility, nutrition care

## Abstract

There is scarce evidence regarding the risk of weight loss and the effect of having registered dietitians (RDs) on staff in rehabilitation wards on weight loss. We aimed to examine the effects of RDs in Kaifukuki (convalescent) rehabilitation wards (KRWs) on the prevention of weight loss in adult patients. Data from 2-year nationwide annual surveys on KRWs in Japan were retrospectively analysed. Weight loss was defined as loss of ≥5% weight during the KRW stay. Risk of weight loss in class 1 KRWs (obligated to provide nutrition care) was compared with that in class 2–6 KRWs (not obligated). Risk of weight loss in class 2–6 KRWs with RDs was compared to those without. Overall, 17.7% of 39,417 patients lost weight. Class 1 KRWs showed a lower risk of weight loss than class 2–6 KRWs (17.3% vs. 18.5%, *p* = 0.003). KRWs with RDs showed a significantly lower incidence of weight loss than those without RDs (16.1% vs. 18.8%, *p* = 0.015). Class 1 KRWs and exclusively staffed RDs were independently associated with lower odds of weight loss (odds ratio = 0.915 and 0.810, respectively). Approximately 18% of KRW patients lost weight, and having RDs on staff can lower the risk of weight loss.

## 1. Introduction

Malnutrition is often seen in older adults and can result in reduced functional capacity. Up to 10.5% of community-dwelling seniors, 7.7% of nursing home residents and 14% of hospitalised patients experience unintentional weight loss [1]. Disabled older adults admitted to rehabilitation hospitals, units or wards are particularly susceptible to worsened nutritional status with a prevalence of 13–30%, and reports suggest a remarkably high proportion (92%) of poor nutritional status in tube-fed patients [2,3,4,5]. Malnutrition may result in muscle attenuation, which can result in a decline in functional capacity in older patients [6]. In fact, undernourished patients show lower activities of daily living (ADL) ability and worse functional recovery, including swallowing function, compared to well-nourished patients [5,7,8]. In addition, previous studies suggest that improvement in nutritional status is significantly associated with better recovery of ADLs in rehabilitation wards [9,10,11]. Nutritional support for older patients, therefore, plays a key role in maximising the effects of rehabilitation therapy.

To prevent malnutrition and regain nutritional status, inpatient rehabilitation should involve nutritional support as part of a multidisciplinary approach. For example, inpatient rehabilitation facilities in the US are obligated to provide a nutritional assessment within 3 days of admission [12]. Another example is the Kaifukuki (convalescent) rehabilitation wards (KRWs) in Japan. The KRW system was established in 2000 under public healthcare policy insurance in Japan. Patients with cerebrovascular disease, musculoskeletal diseases or hospital-associated deconditioning are admitted to the KRWs to maximise ADLs, prevent a bedridden status and promote a successful return home [13]. To achieve this goal, a comprehensive rehabilitation approach is provided by a multidisciplinary team including physicians, nurses, physical therapists (PTs), occupational therapists (OTs), speech–language hearing therapists (STs), medical social workers, care workers, registered dietitians, pharmacists and dental hygienists. Depending on the primary disease, up to 180 min/day for 180 days of rehabilitation therapy provided by the PT, OT and ST is covered by national medical insurance. In 2018, the KRWs were divided into six classes (classes 1 to 6) based on the structure (e.g., number of medical staff on the wards), process (e.g., minimum dose of provided rehabilitation therapy) and outcome measurements (e.g., degree of improvement of ADLs). Of these, only class 1 KRWs established compulsory nutritional assessment by a multidisciplinary team including registered dietitians (RDs), nutrition planning in concordance with the rehabilitation programme, and nutritional monitoring for the patients with nutritional disorders. Additionally, staffing of RDs was recommended in these wards to provide appropriate nutrition care. On the other hand, class 2–6 KRWs did not require RDs or administration of nutrition care, but some KRWs assigned RDs voluntarily. An analysis of a national survey undertaken prior to 2018 shows that the patients in the KRWs staffed with RDs gained significantly more weight than patients in KRWs without RDs [14]. However, as this analysis focused on weight gain in all underweight patients, it was unclear whether all of them were nutritionally supported by RDs.

Loss of body weight is one of the key features of malnutrition; it hampers functional recovery and may be partially preventable with nutritional support [9,11,15]. Since a hypercatabolic status is unlikely to be found in the post-acute phase and most patients undergoing inpatient rehabilitation regain their body weight [15,16,17], it is reasonable to suggest that the loss of body weight in patients during the rehabilitation phase may be attributed to inappropriate nutrition support, unless they receive an energy restrictive diet for obesity. Previous studies have investigated the loss of body weight before admission or post-discharge from rehabilitation facilities [18,19], whereas no studies have focused on weight loss during inpatient rehabilitation. In addition, the effect of having RDs on staff in inpatient rehabilitation facilities for the prevention of weight loss has not been evaluated to the best of our knowledge.

Thus, this study aimed to explore the risk of weight loss in KRWs and examine the effects of having RDs in KRWs on the prevention of weight loss in adult patients in the real world using a nationwide survey. 

## 2. Materials and Methods

### 2.1. Annual Survey

The KRW Association is the sole association of which 84% of the hospitals with KRWs are members [20], and it has been carrying out an annual survey for all member and non-member hospitals with KRWs in Japan. The content of the survey is described elsewhere [13,14]. Briefly, the survey is composed of four parts: (1) hospital-related information (e.g., hospital entity, number of beds, etc.), (2) ward-related information (e.g., number of KRW beds, type and number of healthcare staff working at the KRW, etc.), (3) patient-related information (e.g., age, sex, primary disease, ADL measurements, height, body weight, etc.) and (4) free comments. Patient-related information was usually collected from the patients who were discharged from the KRWs in August and September in each year. The ADLs were measured using the Functional Independence Measure (FIM) [21]. The hospitals were asked to fill out the Excel-based form to collect the data and send the form back to the KRW Association. The response rates of the survey (respondent KRWs/all KRWs in Japan at each period) in 2018 and 2019 were 65.3% (1199/1836) and 62.4% (1194/1914), respectively. The survey results thus represent data from more than 60% of the KRWs in Japan.

### 2.2. Eligibility Criteria and Baseline Characteristics

We obtained the patient-related information that was linked to corresponding hospital- and ward-related information from the dataset of the two-year survey results. All the data from patients aged ≥20 years were included. Data with missing values of height, body weight, FIM, number of RDs on staff and the KRW class were excluded. Data from patients who stayed in the KRWs ≤30 days [14], patients admitted from other KRWs and patients discharged to other KRWs were also excluded because they possibly underwent an incomplete rehabilitation programme. The data of patients who were overweight or obese were also excluded from the analyses of weight loss because, usually, these patients are required to adhere to an energy-restrictive diet for weight loss induction.

Staff RDs were defined by the number of RDs who worked at the ward. They were assigned to one of two categories: (1) the number of RDs who worked at the ward but also completed other tasks such as food service or nutrition management in other wards simultaneously (staffed RD) and (2) the number of RDs who worked at the ward but did not complete other tasks (exclusively staffed RD). In the survey form, ‘staffed RD’ was defined as spending at least 50% of working time in providing nutrition care in the assigned ward, whereas ‘exclusively staffed RD’ was defined as spending ≥80% of working time in providing nutrition care in the ward.

The RDs must adhere to the following regulations when providing nutrition care in KRWs: (1) Obligations; nutritional assessment; participating in preparing a rehabilitation plan; nutritional care planning; nutritional monitoring; nutritional education. (2) Recommendations; nutritional screening; collecting the data on activities of daily living and swallowing function; calculating nutrient requirements; participating in rehabilitation conference; arranging nutritional care plan as a part of discharge planning. Details of the obligations and recommendations for RDs in the KRWs are shown in Table S1. The obligation rules clearly distinguish the quality of clinical practice in the wards with staffed RDs from the quality in those without staffed RDs.

The primary disease/conditions were classified into four categories based on the national healthcare insurance policy: stroke; other neurological diseases/injuries; orthopaedic disease/injuries; and hospital-associated deconditioning. Body mass index (BMI, body weight (kg)/height (m) squared) was categorised into four groups in accordance with the WHO recommendation for the Asian population [22]: underweight, BMI < 18.5 kg/m^2^; normal weight, 18.5 ≤ BMI < 23 kg/m^2^; overweight, 23 ≤ BMI < 27.5 kg/m^2^; obese, BMI ≥ 27.5 kg/m^2^. 

### 2.3. Outcome Measures

The primary outcome of this study was the loss of body weight during the KRW stay, which was defined by at least a 5% decrease in body weight at discharge compared with body weight measured on admission to the KRW. The percentage of weight loss was calculated by subtracting the body weight at discharge from that on admission. Since the upper limit of the length of stay was 180 days (6 months), 5% or more weight loss can be considered as a phenotype of malnutrition [23]. Focusing on the real-world situation, we collected body weights that were measured by non-standardised (depending on facilities’ policy) methods. The accuracy of routinely collected data on body weight towards precisely measured body weight by trained staff was as high as a 0.99 Pearson correlation coefficient for the baseline measurement and 0.96 for change over time [24]. Another study reported the inter-observer reliability for routine measurement of body weight by nurses and research measurement as high as a 0.99 Pearson correlation coefficient [25]. Moreover, a large number of participants (>39,000) were included in this study. Thus, measurement variability for body weight between the hospitals was assumed to be small. The secondary outcome was BMI at discharge. BMI was calculated by dividing the patient’s body weight (kg) at discharge by height (m)^2^; this information was provided as part of the patient-related information in the survey form.

### 2.4. Statistical Tests

All statistical analyses were performed by JMP 11.2.1 software (SAS Japan, Tokyo, Japan). Normally distributed variables were presented as means and standard deviations, whereas skewed variables were expressed as medians and interquartile ranges (IQR). Qualitative variables were reported as numbers (%). The hypothetical test for quantitative variables with normal distribution was performed using a *t*-test, while those with non-normal distribution were performed using a Mann–Whitney U-test. Categorical variables were tested statistically using a chi-squared test or Fisher’s exact test. Significance level was set at *p* < 0.05. Multiple comparisons performed using Bonferroni correction were performed if there was a statistically significant difference for multi-group categorical variables.

Outcome measurements were compared through two separate analyses: (1) Comparison between class 1 KRWs (obligated to nutrition care) and class 2–6 KRWs (not obligated to nutrition care); (2) Comparison between class 2–6 KRWs with and without exclusively staffed RDs. All class 1 KRWs provided the aforementioned nutrition care for relevant patients, and most of them had RDs on staff; therefore, the effectiveness of nutrition care could be tested in Analysis 1. In Analysis 2, the real effect of ‘exclusively staffed RDs’ could be examined because the background of the wards regarding nutrition care other than RDs was similar in classes 2–6. A binary logistic regression analysis was performed to test the association between the incidence of weight loss and the KRW class or having RDs on staff. To adjust for the confounding effect of BMI at discharge, multiple linear regression analysis was performed. Potential confounders for these analyses were selected from the previous literature and biological plausibility [14]: age (continuous), sex (dichotomised: male = 1, female = 0), primary disease (reference: stroke), disease onset to hospital admission duration (continuous), FIM on admission (continuous), body weight on admission (continuous), number of nurses (continuous), and daily rehabilitation dose (min/d, continuous). In Analysis 1, binary logistic regression analysis was performed using the risk of ≥5% weight loss (dichotomised: presence = 1, absence = 0) as a dependent variable and ‘class 1 KRWs’ in addition to the confounders as independent variables. For Analysis 2, binary logistic analysis for weight loss (dependent variable) with ‘exclusively staffed RDs’ (one or more per ward, dichotomised: presence = 1, absence = 0) and the confounders (both independent variables) were implemented. Additionally, multiple linear regression analysis for BMI at discharge (continuous, as dependent variable) with ‘exclusively staffed RDs’ and the confounders were performed in Analysis 2. For multiple linear regression analysis, BMI at admission was used as an independent variable instead of body weight. All multivariable analyses were performed using the forced entry method. 

### 2.5. Ethical Considerations

This study was conducted in accordance with the ethical standards laid down in the 1964 Declaration of Helsinki and its later amendments. This study protocol was approved by the ethical committee of the KRW association (approval number: 2020–027) and the requirement for informed consent was waived because the data on the survey response were all anonymised.

## 3. Results

Table S2 shows the characteristics of the KRWs that responded to the survey. The respondents included 1889 KRWs that were located in 47 prefectures (i.e., covering all prefectures) in Japan. The class 1 group had a high proportion of RDs on staff, more doctors and nurses, and a longer daily rehabilitation dose. The data of a total of 84,263 patients were recorded in the 2018 and 2019 survey datasets and 84,153 patients fulfilled the inclusion criteria of being aged ≥ 20 years. Of these, 13,179 who stayed at the KRWs for ≤30 days, 291 who were admitted from other KRWs or discharged to other KRWs, and 12,958 with missing data were excluded. Finally, 14,288 overweight patients and 4020 obese patients were excluded. Thus, the remaining 39,417 underweight or normal weight patients were analysed. 

Table 1 displays the baseline information of the 39,417 patients. Reasons for admission were stroke (15,274 patients, 38.8%), other neurological disease (3180 patients, 8.1%), orthopaedic diseases (18,032 patients, 45.7%), and hospital-associated deconditioning (2931 patients, 7.4%). According to the BMI on admission, 12,498 (21.7%) patients were classified as being underweight and 26,919 (46.6%) as having normal weight. 

Among them, 25,324 and 14,093 were admitted to class 1 KRWs and class 2–6 KRWs, respectively. The class 1 KRW group included a greater proportion of stroke patients and lower proportion of orthopaedic patients than the class 2–6 group. The former group also showed a shorter length of KRW stay, a lower FIM and a lower proportion of underweight patients than the latter group. Regarding nutrition care, 61.0% of the patients in the class 1 KRW group received nutrition monitoring at least once a week and 24.8% received nutrition counselling during hospitalisation.

Of the 39,417 underweight or normal weight patients, 17.7% of them experienced weight loss overall. The proportion of patients who experienced weight loss was significantly lower in the class 1 KRW group than in the class 2–6 KRW group (17.3% vs. 18.5%, *p* = 0.003).

**Figure 1:**
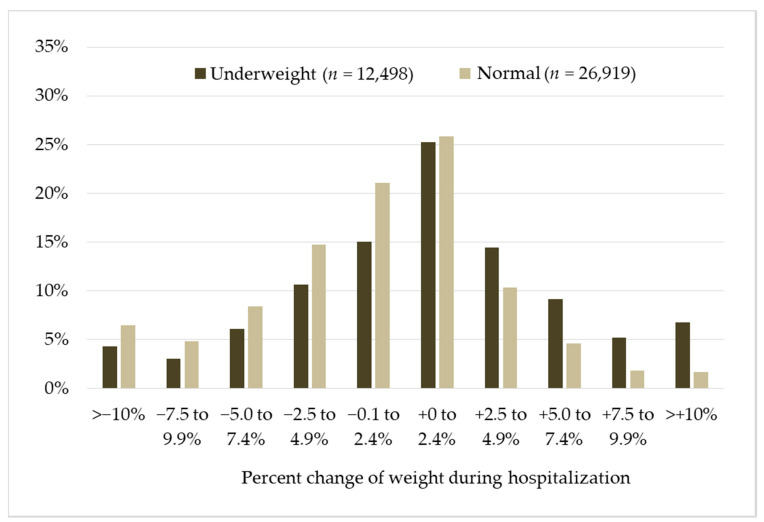
Distribution of percentage of change in body weight during the Kaifukuki (convalescent) Rehabilitation Wards stay in underweight vs. normal weight patients. *Y*-axis indicates proportion of change in weight categories.

Figure 1 illustrates the distribution of percentage of change in body weight during KRW stay. The peaks of weight change distribution in underweight and normal weight patients were similar (0–2.4%), whereas the latter group tended to be more skewed to a negative change than the former. Mean relative change in body weight was significantly different in the underweight and normal weight patients (0.9% vs. −1.5%, *p* < 0.001).

Table 2 shows the proportion of patients who experienced weight loss in KRWs where nutritional care was not provided (classes 2–6) with or without exclusively staffed RDs. As shown below, the proportion of the patients who experienced weight loss was significantly lower in the KRWs with exclusively staffed RDs than those without (16.1% vs. 18.8%, *p* = 0.015).

Table 3 shows the binary logistic regression analysis for weight loss in all participants. Being admitted to a class 1 KRW was independently associated with a lower odds of weight loss (odds ratio (OR) = 0.915; 95% confidence interval (CI), 0.859–0.974; *p* = 0.006). Additionally, age, male sex, diseases other than stroke, days between onset and admission, initial FIM, initial BMI and daily rehabilitation dose were also significantly related to weight loss.

Table 4 shows the binary logistic regression analysis of weight loss in the KRWs where nutrition care was not provided (classes 2–6). Having an exclusively staffed RD (≥1 per ward) was independently associated with lower odds of weight loss (OR = 0.809; 95% CI, 0.683–0.953; *p* = 0.011) as well as male sex, diseases other than stroke, longer duration (days) between onset and admission, higher FIM on admission and greater dose of daily rehabilitation. Conversely, older age and higher body weight were associated with higher odds of weight loss. 

Table S3 shows the results of multiple linear regression analyses for BMI at discharge. Having exclusively staffed RDs (≥1 per ward) (standardised partial regression coefficient (B) = 0.025, 95%CI of B = −0.007–0.058, partial regression coefficient (β) = 0.006, *p* = 0.129) was not independently associated with BMI at discharge. There was a positive relationship between BMI at discharge and other neurological diseases/injuries, days between onset and admission, FIM at admission, BMI at admission and daily rehabilitation dose. In contrast, older age was inversely related to BMI at discharge. 

## 4. Discussion

There were two major findings from this retrospective analysis of a nationwide survey. Firstly, 17.7% of the normal or underweight patients experienced a loss of body weight during their KRW stay. Secondly, the obligation to provide nutrition care and exclusively staffed RDs in the KRW were positively associated with a lower incidence of weight loss.

To date, several studies have reported changes in body weight during rehabilitation, but no studies have focused on patients without obesity [15,16,26,27]. A cohort study conducted in a transitional care unit showed that 15% of patients lost 5% or more of their body weight during their stay in the unit [26]. Another cohort study found that patients in subacute care units showed significantly decreased body weight [27]. In addition, a small study showed that 21% of stroke patients could be described as having ‘cachexia’, which is characterised by a weight loss of ≥5% or more, as well as other features such as decreased muscle mass [15]. Importantly, the mean baseline BMI of the patients in these studies was ≥25 kg/m^2^, suggesting that these studies included obese patients who required weight loss as a part of their treatment. On the other hand, a longitudinal study of stroke patients also showed no weight loss during 104 days of rehabilitation after approximately 14 days from the onset of stroke (mean BMI on admission: 24.5 kg/m^2^, at discharge: 24.8 kg/m^2^) [16]. Our results highlighted that as many as 18% of the underweight and normal weight patients experienced weight loss in the KRWs. Although the reason for weight loss was not clear from our results, older adults often experience decreased body weight due to diseases, dysphagia, dementia and anorexia of ageing, as well as unnecessary caloric restriction [28]. Poor physical function may also cause malnutrition in older adults [29]. Indeed, our results showed that older age and lower FIM were predictive factors for a higher incidence of weight loss. In addition, patients with diseases other than stroke had a lower odds of weight loss. In other words, stroke patients were most likely to experience a decrease in their body weight. Although the reason for this weight loss is unclear, stroke patients have a higher likelihood of developing dysphagia, disabled hand use for eating and muscle atrophy due to hemiparesis than those in patients with other diseases; this may partially explain our results. Interestingly, a higher rehabilitation dose was correlated with a lower odds of weight loss. This finding is consistent with our previous study [14]. As low-intensity exercise might stimulate appetite [30], a sufficient rehabilitation dose is recommended to stimulate food intake and prevent weight loss. 

In our analyses, more than 80% of the class 1 KRWs that were obligated to provide nutrition care had RDs on staff, and they showed a significantly lower incidence of weight loss than the class 2–6 KRWs. More importantly, having exclusively staffed RDs in the class 2–6 KRWs was negatively associated with weight loss. These findings are consistent with previous studies [14,31] and suggest that RDs may prevent patients in rehabilitation wards from unnecessary weight loss. In general, RDs perform nutrition assessment, nutrition diagnosis, nutrition intervention (e.g., recommendation for nutritional prescription, nutritional education) and nutrition monitoring (known as nutrition care process) in clinical practice [32]. Planning nutrition care based on the underlying disease, functional goals, patient-centred goals and timely monitoring are the four core elements to maintain nutritional status as they are linked to increased energy and preserved body weight [14,33]. In addition, RDs working in class 1 KRWs were mandated to participate in nutritional assessment, planning and monitoring for malnourished patients. It is therefore reasonable that the assignment of RDs to the KRWs can be effective for improving or maintaining nutritional status in rehabilitative inpatients. However, our study also showed that 19.5% of the underweight or normal weight patients experienced ≥5% weight loss, despite there being RDs assigned to the KRW. As we could not collect the details of the actual practice that the RDs provided to the patients from the datasets, we could not ascertain how many patients truly received appropriate nutrition care. To solve this problem, the KRW association issued clinical practice guidebooks (Kaifukuki Rehabilitation Ward Association Nutrition Committee: Vade mecum for registered dietitians in Kaifukuki Rehabilitation Wards) to each RD in member hospitals and provided 1-day courses twice a year to promote learning. Further investigations are needed to clarify the effectiveness of clinical nutrition practice in inpatient rehabilitation services.

One of the strengths of our study was its analysis of a large number of samples in the real-world setting from the national survey. To the best of our knowledge, this is the first study to examine weight loss during inpatient rehabilitation using nationwide samples. As described, the original datasets covered 62–65% of all KRWs in Japan and there seems to be a small amount of bias regarding regional variety due to the location of the KRW. Additionally, Japan applied universal healthcare insurance coverage for all people living in Japan [34], and consequently, healthcare costs for all patients in KRWs are covered by public healthcare insurance. Our results may, therefore, represent the real status of the whole country.

However, there were some limitations to this study. First, the pathophysiology of malnutrition, as well as the actual nutritional status of patients and details of the nutrition care provided, could not be analysed because the original datasets did not contain this information. Because the current consensus on the definition of malnutrition relies on both phenotype (e.g., weight loss) and aetiology (e.g., decreased nutrient intake) [23], actual changes in the nutritional status of the study patients and the effects of nutrition care remain to be resolved. Second, as the KRW is a unique rehabilitation system in Japan, the generalisability of the results for other countries is unclear. However, our findings can, at least partially, be applied to inpatient rehabilitation services in other countries because weight loss during rehabilitation is likely to be a common issue in many countries [26,27,28]. Third, the type of nutritional care that was provided to the patients remains uncertain. Further investigation is required to determine the best nutrition practice that could prevent the loss of body weight. Forth, information on the actual activities of RDs was not available in the survey form. Although there are certain regulations and recommendation for RDs’ practice, we could not identify any specific practice of those RDs who contributed to a lower likelihood of patients’ body weight loss. Lastly, the procedures followed for the measurement of body weight were not standardised because all data were retrospectively collected based on routine practice in each KRW. However, previous reports indicated a strong correlation between routinely collected data on body weight and the data on body weight measured by standardised methods [24,25]. Moreover, numerous studies that were published recently have reported on real-world (unstandardised) body weight [14,35,36,37,38,39]. Thus, the data on body weight reported in our study may be reliable and represent a ‘real-world situation’ that is not fully standardised for body weight measurements. Our results will guarantee further prospective study with a standardised method for data collection to investigate the essential role of RDs in in-patient rehabilitation facilities.

## 5. Conclusions

This retrospective analysis of a nationwide survey of KRWs in Japan revealed that 17.7% of underweight or normal weight patients lost more than 5% of their body weight during hospitalisation. In addition, the KRWs obligated to provide nutrition care and with exclusively staffed RDs were able to significantly reduce the incidence of weight loss. Further research is needed to clarify the actual changes in nutritional status during the KRW stay, and the effect of RD practice on improvements in nutritional disorders.

## Figures and Tables

**Table 1:** Characteristics of the 39,417 underweight and normal weight patients admitted to different classes of Kaifukuki (convalescent) Rehabilitation Wards.

Factor	All	Class 1 KRWs *	Class 2–6 KRWs ^†^	*p* Value
Number of individuals, (%)	39,417 (100)	25,324 (64.2)	14,093 (35.8)	
Age, years, median (IQR)	81 (73–87)	81 (72–87)	82 (74–87)	<0.001 ^1^
Female, *n* (%)	23,225 (58.9)	14,714 (58.1)	8511 (60.4)	<0.001 ^2^
Disease, *n* (%)				<0.001 ^2^
Stroke	15,274 (38.8)	10,328 (40.8)	4946 (35.1)	
Other neurological diseases	3180 (8.1)	2223 (8.8)	957 (6.8)	
Orthopaedic diseases	18,032 (45.7)	10,965 (43.3)	7067 (50.1)	
Hospital-associated deconditioning	2931 (7.4)	1808 (7.1)	1123 (8.0)	
Days between onset and admission, median (IQR)	23 (15–35)	23 (15–34)	23 (15–36)	0.006 ^1^
Length of hospitalisation, median (IQR)	75 (53–91)	74 (53–91)	77 (53–90)	<0.001 ^1^
FIM, median (IQR)				
Admission	62 (41–83)	62 (40–81)	64 (42–85)	<0.001 ^1^
Discharge	97 (65–115)	98 (66–115)	96 (64–114)	<0.001 ^1^
Gain	24 (12–37)	26 (13–38)	22 (10–34)	<0.001 ^1^
Discharge destination, n (%)				<0.001 ^2^
Home	24,982 (63.4)	16,149 (63.7)	8833 (62.7)	
Death	196 (0.5)	105 (0.4)	91 (0.7)	
Acute care hospital	2758 (7.0)	1701 (6.7)	1057 (7.4)	
Long-term care hospital	2097 (5.3)	1405 (5.6)	692 (4.9)	
Long-term care facilities	9384 (23.8)	5964 (23.6)	3420 (24.3)	
BMI, n (%)				<0.001 ^2^
Underweight (<18.5 kg/m^2^)	12,498 (31.7)	7816 (30.9)	4682 (33.2)	
Normal (18.5 to <23 kg/m^2^)	26,919 (68.3)	17,508 (69.1)	9411 (66.8)	
Weight loss, n (%)	6989 (17.7)	4382 (17.3)	2607 (18.5)	0.003 ^1^

^1^ Mann–Whitney U-test, ^2^ Chi-square test. IQR, interquartile range; FIM, Functional Independence Measure; BMI, body mass index; KRWs, Kaifukuki Rehabilitation Wards. * Obligated to provide nutrition care, ^†^ Not obligated to provide nutrition care.

**Table 2:** Characteristics of the 14,093 underweight and normal weight patients admitted to class 2–6 KRWs.

Factor	All	KRWs with Exclusively Staffed RDs *	KRWs without Exclusively Staffed RDs	*p* Value
Number	14,093	1392 (9.9)	12,701 (90.1)	
Age, median (IQR)	82 (74–78)	82 (74–88)	82 (74–87)	0.428 ^1^
Female, n (%)	8511 (60.4)	852 (61.2)	7659 (60.3)	0.512 ^2^
Disease, n (%)				0.040 ^2^
Stroke	4946 (35.1)	475 (34.1)	4471 (35.2)	
Other neurological diseases/injuries	957 (6.8)	88 (6.3)	869 (6.8)	
Orthopaedic diseases/injuries	7067 (50.1)	691 (49.6)	6376 (50.2)	
Hospital-associated deconditioning	1123 (8.0)	138 (10.0)	985 (7.8)	
FIM at admission, median (IQR)	64 (42–85)	62 (41–83)	64 (42–85)	0.020 ^1^
Weight loss, n (%)	2607 (18.5)	224 (16.1)	2383 (18.8)	0.015 ^2^

^1^ Mann–Whitney U-test, ^2^ Chi-square test; IQR, interquartile range; FIM, Functional Independence Measure; KRWs, Kaifukuki Rehabilitation Wards; RD, registered dietitian. * RDs who worked at the ward but did not complete other tasks

**Table 3:** Binary logistic regression analysis of weight loss * in all KRWs.

Factor	OR	95%CI		*p* Value
		Lower	Upper	
Age	1.017	1.014	1.020	<0.001
Sex, male	0.703	0.655	0.755	<0.001
Disease				
Stroke	Reference			
Other neurological diseases/injuries	0.682	0.603	0.769	<0.001
Orthopaedic diseases/injuries	0.827	0.772	0.887	<0.001
Hospital-associated deconditioning	0.889	0.794	0.994	0.039
Days between onset and admission	0.993	0.991	0.995	<0.001
FIM at admission	0.984	0.983	0.985	<0.001
Body weight at admission	1.051	1.047	1.056	<0.001
Number of nurses	0.998	0.992	1.004	0.529
Daily rehabilitation dose (min/d)	0.998	0.997	0.999	<0.001
Class 1 KRWs ^†^	0.915	0.859	0.974	0.006

OR, odds ratio; CI, confidence interval; FIM, Functional Independence Measure; KRWs, Kaifukuki Rehabilitation Wards. * defined as ≥5% loss during the KRW stay, ^†^ obligated to provide nutrition care, R^2^ = 0.044.

**Table 4:** Binary logistic regression analysis of weight loss * in the KRWs where nutrition care was not provided (classes 2–6).

Factor	OR	95%CI		*p* Value
		Lower	Upper	
Age	1.016	1.011	1.021	<0.001
Sex, male	0.650	0.577	0.731	<0.001
Disease				
Stroke	Reference			
Other neurological diseases/injuries	0.745	0.603	0.915	0.005
Orthopaedic diseases/injuries	0.785	0.700	0.881	<0.001
Hospital-associated deconditioning	0.823	0.683	0.987	0.036
Days between onset and admission	0.995	0.991	0.998	0.001
FIM at admission	0.983	0.981	0.985	<0.001
Body weight at admission	1.056	1.048	1.064	<0.001
Number of nurses	1.000	0.991	1.010	0.886
Daily rehabilitation dose (min/d)	0.998	0.996	0.999	<0.001
Exclusively staffed registered dietitian (≥1 per ward)	0.810	0.683	0.955	0.012

OR, odds ratio; CI, confidence interval; FIM, Functional Independence Measure. * defined as ≥5% loss during the KRW stay. R^2^ = 0.051.

## Data Availability

Data sharing not applicable.

## Supplementary Materials

The following are available online at https://www.mdpi.com/article/10.3390/healthcare9060753/s1, Table S1: Obligations and recommendations for registered dietitians (RDs) in the Kaifukuki (convalescent) rehabilitation wards, Table S2: Summary of the characteristics of the 1889 Kaifukuki (convalescent) Rehabilitation Wards that responded to the survey, Table S3: Multiple linear regression analysis of BMI at discharge in the KRWs where nutrition care was not provided (class 2–6).
