# Supplementary material for: Risk of Weight Loss in Adult Patients and the Effect of Staffing Registered Dietitians in Kaifukuki (Convalescent) Rehabilitation Wards: A Retrospective Analysis of a Nationwide Survey
Source: Healthcare (Basel). 2021 Jun 18;9(6):753. doi: 10.3390/healthcare9060753 (PMC8235006; doi:10.3390/healthcare9060753)
Supplement: Supplementary file 1 [file healthcare-09-00753-s001.zip › healthcare-1197164-supplementary.pdf]

Table S1. Obligations and recommendations for registered dietitians (RDs) in the Kaifukuki (convalescent) rehabilitation wards.

**Obligations** [1]
RDs must include the nutritional status of all patients while planning the rehabilitation programs. Nutrition-related information must be included on the form of the rehabilitation program as follows: height, body weight, body mass index, nutritional status, total energy and protein requirements, provided energy and protein, type of nutrition support (oral intake, oral nutritional supplements, enteral nutrition via naso-gastric tube or gastrostomy, partial or total parenteral nutrition), and need for texture-modified diet.
RDs must assess and re-assess the nutritional status of patients at admission and on a regular basis, and if applicable, must arrange a nutrition care plan in collaboration with the medical doctor, nurses, and other healthcare staff.
The nutritional status of patients with malnutrition, at risk of malnutrition or other statuses that require specific nutritional support must be monitored at least once a week, and should be provided appropriate nutritional support in consideration of the nutrition monitoring, to regain a healthy nutritional status.
RDs must make a nutritional care plan for all patients who need nutritional support.
RDs must provide nutritional education to relevant patients.
**Recommendations (apart from the obligations)** [2]
RDs should apply appropriate nutrition screening tools and construct a nutrition screening system in collaboration with the nursing and other healthcare staff.
RDs should collect data on activities of daily living of the patients.
RDs should collect the data on the swallowing function of patients.
RDs should calculate the energy, protein and other relevant nutritional requirements for each patient.
RDs should participate in rehabilitation conferences to share and discuss nutritional status, goals, and care plans for patients.
RDs should arrange the nutritional care plan in consideration of the nutritional status, living conditions, willingness, swallowing function, ability to perform daily activities, and socio-economic status of the patients.

[1] Referred by the public healthcare insurance policy in Japan. 2 Referred by "Vade mecum for registered dietitians in Kaifukuki Rehabilitation Wards" edited by the Kaifukuki Rehabilitation Ward Association Nutrition Committee.

Table S2. Summary of the characteristics of the 1889 Kaifukuki (convalescent) Rehabilitation Wards that responded to the survey.

**Factor**	**All**	**KRWs Class 1 ***	**KRWs Class 2-6 †**	***p* Value**
Number of wards, (%)	1889	1150 (60.9)	739 (39.1)	—
Number of covered prefectures, (%)	47 (100)	47 (100)	47 (100)	—
Staffed RD, (%)	1188 (62.9)	840 (70.7)	348 (29.3)	<0.001 [1]
Exclusively staffed RD, (%)	296 (15.7)	236 (20.5)	60 (8.1)	<0.001 [1]
Number of physicians, median (IQR)	1 (1–1)	1 (1–1)	1 (1–1)	0.007 [2]
Number of nurses, median (IQR)	19 (16–23)	21 (18–24)	17 (14–20)	<0.001 [2]
Daily rehabilitation dose (min/d), median (IQR)	134 (106–158)	144 (116–162)	118 (90–146)	<0.001 [2]

[1] Chi-square test, [2] Mann–Whitney U-test. IQR, interquartile range; RD, registered dietitian; KRWs Kaifukuki Rehabilitation Wards. * obligated to provide nutrition care, † Not obligated to provide nutrition care.

Table S3. Multiple linear regression analysis of BMI at discharge in the KRWs where nutrition care was not provided (class 2–6).

Factor	B	95%CI of B		β	*p* Value
		Lower	Upper		
Age	−0.007	−0.009	−0.005	−0.033	<0.001
Sex, male	0.002	−0.019	0.023	0.001	0.860
Disease					
Stroke	Reference				
Other neurological diseases/ injuries	0.107	0.047	0.167	0.026	0.001
Orthopaedic diseases/ injuries	−0.006	−0.042	0.030	−0.002	0.736
Hospital-associated deconditioning	−0.015	−0.070	0.041	−0.004	0.607
Days between onset and admission	0.004	0.002	0.005	0.024	< 0.001
FIM at admission	0.005	0.004	0.006	0.056	< 0.001
BMI at admission	0.875	0.866	0.883	0.867	< 0.001
Number of nurses	0.004	0.000	0.008	0.008	0.066
Daily rehabilitation dose (min/d)	0.001	0.001	0.002	0.023	< 0.001
Exclusively staffed registered dietitian (≥1 per ward)	0.025	-0.007	0.058	0.006	0.128

BMI, body mass index; B, standardised partial regression coefficient; β, partial regression coefficient; CI, confidence interval; FIM, Functional Independence Measure, $R^2 = 0.783$.
